# Thyroid-associated ophthalmopathy and ferroptosis: a review of pathological mechanisms and therapeutic strategies

**DOI:** 10.3389/fimmu.2024.1475923

**Published:** 2024-12-06

**Authors:** Chao Ma, Haoyu Li, Shuwen Lu, Xian Li

**Affiliations:** ^1^ Department of Ophthalmology, the First Affiliated Hospital of Zhengzhou University, Zhengzhou, Henan, China; ^2^ Department of Ophthalmology, the Second Xiangya Hospital of Central South University, Changsha, Hunan, China; ^3^ Hunan Clinical Research Centre of Ophthalmic Disease, Changsha, Hunan, China; ^4^ Department of Ophthalmology, the First Affiliated Hospital of Henan University of Chinese Medicine, Zhengzhou, Henan, China; ^5^ Division of Pharmacy and Optometry, School of Health Sciences, Faculty of Biology, Medicine and Health, The University of Manchester, Manchester, United Kingdom

**Keywords:** thyroid-associated ophthalmopathy, ferroptosis, oxidative stress, inflammation, fibrosis, therapeutic strategies

## Abstract

Thyroid-associated ophthalmopathy (TAO) is an inflammatory orbital disease associated with autoimmune thyroid disorders. Owing to the ambiguous nature of the pathogenesis, contemporary pharmacological treatment strategies predominantly involve the use of glucocorticoids and immunosuppressants. However, the adverse effects associated with these agents in clinical practice necessitate further investigation into the disease’s pathogenesis and the identification of novel therapeutic targets and pharmacological interventions. Recent studies suggest that ferroptosis, a novel form of regulated cell death, may play a role in TAO pathogenesis. This review aims to explore the involvement of ferroptosis in TAO and evaluate its potential as a therapeutic target. Key topics include the epidemiology, clinical manifestations, and pathophysiology of TAO, along with the molecular mechanisms of ferroptosis. Evidence supporting ferroptosis in TAO and the therapeutic implications of targeting this pathway are also discussed, alongside challenges and future directions in this emerging research area.

## 1 Introduction

Thyroid-associated ophthalmopathy (TAO), also known as Graves’ orbitopathy, is a complex autoimmune disorder that primarily impact the orbital and periorbital tissues (1). TAO represents the most common extrathyroidal manifestation of Graves’ disease (GD), occurring in approximately 25% of individuals diagnosed with GD (2). TAO is characterized by orbital inflammation and tissue remodeling, leading to symptoms that range from mild discomfort to severe vision impairment and disfigurement (3). The incidence of TAO was reported to be between 0.54 and 0.9 cases per 100,000 per year in males, and between 2.67 and 3.3 cases per 100,000 per year in females. The majority of patients experienced mild TAO, whereas those with moderately severe and severe TAO constituted 5-6% of the total patient population (4). The pathophysiology of TAO involves a complex interplay of immune cells, cytokines, and autoantibodies (5). Ferroptosis, a novel mechanism of cell death, may play a role in the onset and progression of TAO.

Ferroptosis, a regulated form of cell death driven by iron-dependent lipid peroxidation, has emerged as a significant factor in various diseases, notably cancer and neurodegeneration. Its role in TAO, however, is a relatively new area of investigation. Unlike apoptosis or necrosis, ferroptosis is uniquely dependent on iron and reactive oxygen species (ROS) (6). Investigating the role of ferroptosis in TAO opens new avenues for understanding TAO’s pathophysiology and potential treatment therapies. Owing to the ambiguous pathogenesis of TAO, the primary pharmacological interventions remain glucocorticoids and immunosuppressants (7). Nevertheless, prolonged administration of glucocorticosteroids may result in adverse effects, including fat redistribution, osteoporosis, and femoral head necrosis (8). Similarly, immunosuppressants can adversely affect the reproductive system, among other potential complications (9). Consequently, it is imperative to investigate the pathogenesis and develop novel therapeutic strategies for the treatment of TAO. Selenium is a well-established inhibitor of ferroptosis, and supplementation with selenium has demonstrated positive effects on quality of life and long-term prognosis in patients with mild TAO (10, 11). Additionally, teprotumumab, a human monoclonal antibody that inhibits the insulin-like growth factor 1 receptor (IGF1R), has been shown to reverse the metabolic switch and enhance sensitivity to ferroptosis (12). This drug has recently received approval from the U.S. Food and Drug Administration for the treatment of active, moderate-to-severe TAO (13). Therefore, ferroptosis may be involved in the occurrence and development of TAO and may become a new therapeutic target. To understand the mechanism of ferroptosis in TAO, we will first review the epidemiology and clinical features of TAO, then delve into its immunological background and the molecular mechanisms of ferroptosis. Following this, we will examine evidence linking ferroptosis to TAO and discuss the therapeutic potential of ferroptosis inhibitors. Lastly, we will address the challenges and future directions in this emerging field. In summary, this article examines the role of ferroptosis in TAO, assesses its viability as a therapeutic target, investigates therapeutic agents that modulate ferroptosis, and addresses potential challenges associated with these interventions.

## 2 The Relationship between TAO and ferroptosis

### 2.1 Molecular mechanism of TAO

TAO is characterized by a complex molecular mechanism involving interactions at various cellular and molecular levels and is classified as an autoimmune disease. In the context of abnormal thyroid function, the immune system generates antibodies targeting the thyroid-stimulating hormone receptor (TSHR) (14). These antibodies target thyroid cells and influence eye immune and cellular responses. Binding of TSHR antibodies activates receptors via the G protein-coupled signaling pathway, stimulating adenylate cyclase, raising intracellular adenosine monophosphate levels, and triggering cell proliferation and collagen synthesis (15). In TAO, elevated IGF-1R expression interacts with TSHR antibodies, leading to ocular tissue growth and glycosaminoglycan deposition. IGF-1 activates the PI3K/Akt pathway, enhancing cell survival and proliferation (16). Cytokines and inflammatory factors regulate TAO development. TNF-α stimulates fibroblast growth, extracellular matrix production, and inflammatory cell migration. IL-1 and IL-6 enhance inflammation by promoting lymphocyte and macrophage chemotaxis (17). In the context of fibrosis, TGF-β is crucial in TAO-related fibrosis, as it activates fibroblasts and boosts collagen production via the Smad signaling pathway, leading to increased extracellular matrix components like collagen, fibronectin, and glycosaminoglycans (18). Furthermore, in TAO, there is an upregulation of collagen synthesis accompanied by a reduction in its degradation, resulting in collagen accumulation. This alteration is associated with an imbalance in cytokine levels and the regulation of matrix metalloproteinases (19). Regarding immune cell infiltration, the activation and infiltration of macrophages can exacerbate inflammatory responses, thereby facilitating the destruction and fibrosis of ocular tissues (20). The molecular mechanisms underlying TAO are intricate, encompassing the interplay of various cell types, signaling pathways, and molecular mediators. Elucidating and broadening our comprehension of these mechanisms is of paramount importance for the development of novel therapeutic strategies.

### 2.2 Ferroptosis and oxidative stress in TAO

Iron, a crucial metallic element, plays a vital role in numerous cellular biological processes, such as oxygen transport and electron transfer (21). Nonetheless, excessive intracellular iron concentrations can facilitate the production of reactive oxygen species (ROS), which are highly reactive molecules that can inflict damage on cellular structures, including DNA, proteins, and lipids (22). Ferroptosis is a unique form of regulated cell death characterized by iron-dependent lipid peroxidation and ROS accumulation (23). Contrasting with apoptosis and necrosis, ferroptosis is primarily driven by a collapse in cellular antioxidant defenses, specifically through glutathione (GSH) depletion and glutathione peroxidase 4 (GPX4) inactivation (24, 25). This process initiates with the accumulation of unbound iron, which catalyzes the formation of highly reactive hydroxyl radicals via the Fenton reaction. These radicals subsequently induce lipid peroxidation, disrupting cellular membranes and ultimately leading to cell death. Morphologically, ferroptotic cells exhibit condensed mitochondria with increased membrane density and reduced or absent cristae—distinct features that differentiate ferroptosis from apoptosis and necrosis (26, 27).

Oxidative stress plays a pivotal role in the pathogenesis of TAO. In affected individuals, elevated ROS levels or a reduced antioxidant ability can lead to oxidative damage in cellular membranes, lipid peroxidation, and DNA oxidation. This stress response is characterized by significantly increased levels of lipid peroxides, superoxide dismutase (SOD), glutathione reductase, and glutathione peroxidase within the orbital connective tissue, alongside a decrease in glutathione, a major antioxidant (28). Furthermore, modulation of the nuclear factor erythroid 2-related factor 2 (Nrf2)/extracellular signal-regulated kinases (ERK)/heme oxygenase-1 (HO-1) signaling pathway has shown potential in mitigating oxidative stress in orbital fibroblasts (29). In addition, increased expression of endoplasmic reticulum stress (ER)-related genes, such as activating transcription factor 6, phospho-ERK, and inositol-requiring enzyme 1α, has been observed in the orbital tissues of TAO patients compared to controls (30). Inhibition of phospho-ERK has been shown to reduce oxidative stress and lipid synthesis in TAO orbital fibroblasts (31). Epigenetic regulators, particularly histone deacetylases, play a pivotal role in modulating immune response and fibrosis in TAO (32). Additionally, empirical studies have provided evidence that using RNA aptamers (e.g., CD40Apt) to inhibit the CD40-CD40L signaling pathway can reduce the expression of CD40, collagen I, transforming growth factor beta (TGF-β), and alpha smooth muscle actin (α-SMA) in the orbital muscles and adipose tissue in murine models (33).

Oxidative stress plays a central role in triggering ferroptosis. The fundamental molecular mechanism involves an imbalance between oxidative damage and antioxidant defenses, particularly affecting lipid membranes (34). This process is intensified by iron-catalyzed lipid peroxide formation, which further disrupts the antioxidant defense system—primarily through GSH depletion and GPX4 inhibition—thereby exacerbating oxidative stress and promoting ferroptosis (35). Accumulated lipid peroxides amplify this oxidative stress, creating a self-perpetuating cycle that drives cell death (35). Several antioxidant pathways modulate ferroptosis, including the Xc system - GPX4 pathway, the ferroptosis suppressor protein 1-coenzyme Q10 pathway, the GTP cyclohydrolase 1-tetrahydrobiopterin pathway, and the dihydroorotate dehydrogenase-coenzyme Q10 pathway (36, 37). These pathways are crucial for synthesizing antioxidants like GSH, coenzyme Q10H2, and tetrahydrobiopterin, which collectively mitigate intracellular oxidative stress and prevent ferroptosis (38, 39). During ferroptosis, cellular redox homeostasis is disrupted, with diminished antioxidants such as GSH and GPX4 and increased levels of pro-oxidants, including divalent iron ions and lipid ROS (40). The mitochondria, a primary source of intracellular ROS, are notably implicated in ferroptosis (41). Consequently, oxidative stress represents a significant mechanism contributing to ferroptosis, which plays a role in the onset and progression of TAO. Mitigating oxidative stress or inhibiting ferroptosis may offer multifaceted approaches to controlling the advancement of TAO.

The interplay between ferroptosis and mitochondrial biogenesis is particularly noteworthy. Nrf2, a critical regulator in ferroptosis, influences mitochondrial biogenesis by modulating genes such as peroxisome proliferator-activated receptor gamma coactivator 1-alpha, Nrf1 and Nrf2, transcription factor A mitochondrial, and other mitochondrial genes (42). Furthermore, Nrf2 mediates the regulation of mitochondrial fission, fusion, and autophagy. In conclusion, an aberrant redox system marked by excessive oxidants and depleted antioxidants leads to lipid peroxide accumulation, inducing ferroptosis. This underscores the therapeutic potential of targeting redox homeostasis to prevent ferroptosis-related diseases (43). Moreover, ROS generated during inflammatory responses can induce cellular damage and play a pivotal role in activating ferroptosis. Studies have demonstrated that markers of oxidative stress are elevated in the orbital tissues of TAO patients, indicating that ROS accumulation may instigate ferroptosis in orbital fibroblasts (44). It is important to note that ferroptosis is distinct from apoptosis and necrosis, being driven specifically by iron-catalyzed lipid peroxidation. The involvement of ferroptosis in TAO suggests that targeting oxidative stress and iron metabolism could be effective therapeutic strategies for managing TAO (45). These findings imply that oxidative stress and ferroptosis contribute to TAO pathogenesis by promoting inflammation and tissue remodeling. If oxidative stress and ferroptosis can be jointly suppressed, it may have a better effect.

### 2.3 Ferroptosis and inflammation in TAO

The pathogenesis of TAO involves a complex interplay among immune cells and cytokines. CD4^+^ T cells, particularly Th1 and Th17 subsets, are central to orchestrating the immune response in TAO. These cells secrete pro-inflammatory cytokines such as interferon-gamma (IFN-γ), interleukin-1 beta (IL-1β), and interleukin-17 (IL-17), which stimulate the activation and proliferation of orbital fibroblasts and adipocytes (46). Additionally, B cells contribute to disease progression by producing autoantibodies against the thyroid stimulating hormone (TSH) receptor and insulin-like growth factor 1 receptor. Macrophages and dendritic cells also participated in the inflammatory milieu of TAO, releasing cytokines like tumor necrosis factor-alpha (TNF-α) and interleukin-6 (IL-6), which further amplify tissue inflammation and fibrosis (47). This dysregulation of immune cells and cytokines establishes a pro-inflammatory environment that drives the pathogenesis of TAO. Studies have shown that tea-derived polyphenols can suppress IL-6, IL-1β, and monocyte chemoattractant protein-1 synthesis in TAO orbital fibroblasts via modulation of the nuclear factor kappa B (NF-κB)/NLR family pyrin domain containing 3 (NLRP3) pathways, thereby reducing inflammation triggered by lipopolysaccharide (LPS) (48). In an *in vitro* model of TGF-β-induced orbital fibroblast activation, inhibition of TGF-β reduced levels of α-SMA, type I collagen, Timp-1, and vimentin, and decreased TGF-β-induced phosphorylation of ERK, p38, JNK, and NF-κB (49). Furthermore, the Janus kinase (JAK)-signal transducer of activation (STAT) signaling pathway has been identified as critical for regulating orbital inflammation in TAO (50, 51). Similarly, activation of the mitogen-activated protein kinases (MAPK)/ERK1/2 signaling pathway promotes orbital fibroblast proliferation and differentiation, exacerbating inflammatory response in TAO orbital tissues (52). TSH has been shown to induce interleukin-1 receptor antagonist (IL-1RA) production in fibroblasts and orbital fibroblasts through the phosphoinositide 3-kinase (PI3K)/protein kinase B (AKT) pathway (53). Inhibition of PI3K or AKT using small molecule inhibitors, or silencing their expression with small interfering RNA, attenuates TSH’s effects (54). DNA methylation is also implicated in regulating inflammatory receptors and basal metabolic rate in TAO (55), while decreased histone deacetylase 2 expression in ocular tissues enhances T cell proliferation and inflammation in thyroid eye disease (56).

Ferroptosis, characterized by disruptions in redox homeostasis and increased lipid peroxidation, can stimulate inflammatory cells and pathways, leading to pro-inflammatory cytokine production that, in turn, intensifies intracellular oxidative stress and further promotes lipid peroxidation (57). Arachidonic acid (AA) serves as a principal constituent of cell membrane lipids and is metabolized into active pro-inflammatory mediators through three major metabolic pathways. Specifically, AA is converted to prostaglandins via the cyclooxygenase pathway, to leukotrienes and lipoproteins via the lipoxygenase pathway, and to epoxyeicosatrienoic acid and hydroxyeicosatetraenoic acid via the cytochrome P450 pathway (58). Recent studies have elucidated the role of ferroptosis in the NF-κB signaling pathway, where phosphorylation of p65 augments the transcription of lipocalin-2. This reduces extracellular iron uptake, decreasing susceptibility to ferroptosis in hepatic cells (59). In smooth muscle cell studies, NF-κB pathway activation was associated with increased pro-inflammatory cytokine release, including TNF, C-X-C motif ligand (CXCL) 1, CXCL8, and colony-stimulating factor 2. Conversely, treatment with ferroptosis inhibitors reduced the release of TNF-α, IL-1β, and IL-6, thereby alleviating liver damage in experimental models (60). Similarly, dimethyl fumarate, an activator of Nrf2, regulates IκBα and inhibits NF-κB signaling pathway, which, in turn, enhances the transcription of ferroptosis-related protective factors such as heme oxygenase 1, NAD(P)H quinone oxidoreductase 1, and GPX4, therefore providing defense against oxidative stress and ferroptosis (61).

IFN-γ is a critical cytokine in host immunity against tumors, enhancing the susceptibility of tumor cells to ferroptosis through the JAK-STAT signaling pathway (62). IFN-γ therapy has been shown to decelerate the growth of transplanted tumors by promoting lipid oxidation within the host (63). Chromatin immunoprecipitation assays reveal that IFN-γ facilitates the binding of STAT1 to the promoter of solute carrier family 7-member 11 (SLC7A11). Furthermore, the absence of STAT1 abrogates the effects of IFN-γ on ferroptosis and lipid peroxidation (64). In addition to its role in tumor growth, IFN-γ also suppresses the synthesis of GSH through the JAK1/2-STAT1-SLC7A11 signaling pathway, mediating ferroptosis in retinal pigment epithelial cells, which has been linked to *in vivo* macular degeneration (65).

Similarly, the activation of inflammation through the MAPK pathway is pivotal in triggering ferroptosis (66). In a neonatal rat model subjected to hypoxia-ischemia, the activation of the TLR4-p38 MAPK pathway has been observed (67). This activation increases the production of pro-inflammatory cytokines such as IL-1β, IL-6, and IL-18, while simultaneously inhibiting the expression of SLC7A11 and GPX4 (68). This sequence of molecular events result in neuroinflammation and ferroptosis (69). Additionally, ERK serves as a mediator of the inflammatory response and ferroptosis. Cadmium telluride quantum dot exposure, for example, enhances iron autophagy through the Nrf2-ERK pathway, leading to iron release from labile iron pools, which in turn triggers macrophage ferroptosis and inflammation (70). In conclusion, inflammation not only directly impacts orbital tissue but also exacerbates damage in patients with TAO by serving as an inducing factor for ferroptosis. Consequently, targeting ferroptosis in therapeutic interventions may further mitigate orbital inflammatory responses and confer a protective effect.

### 2.4 Ferroptosis and fibrosis in TAO

The intricate interplay of tissue expansion, remodeling, and fibrosis in TAO significantly contributes to the onset and progression of the disease (71). The pathophysiological mechanisms underlying TAO encompass the infiltration of various immune cells, including CD4^+^ and CD8^+^ T cells, mast cells, and B cells, into orbital tissue. This infiltration results in dysregulated immune responses and abnormal accumulation of hyaluronic acid and glycosaminoglycans (72). TGF-β is central to the development of orbital fibrosis in TAO, mediating tissue fibrosis through both classical and non-classical suppressor of mothers against decapentaplegic (SMAD) signaling pathways (73). Matrix metalloproteinases facilitate the cleavage of latent TGF-β binding protein and latent associated peptide, thereby releasing activated TGF-β, which subsequently engages with TGF-β receptors (74). This receptor interaction induces the phosphorylation of SMAD2/3, resulting in the formation of a complex with SMAD4. The SMAD2/3/4 complex translocates to the nucleus to drive myofibroblast transdifferentiation and extracellular matrix (ECM) production (75). In non-SMAD pathways, TGF-β activates MAPK, PI3K, and Rho-like GTPases, further promoting ECM synthesis and fibroblast transdifferentiation in TAO orbital tissues (76). Inhibiting the p38 or JNK pathways may offer therapeutic potential in treating orbital fibrosis.

Smoking significantly exacerbates orbital fibrosis in TAO patients. IL-1 and ROS produced from cigarette smoke synergistically increase hyaluronic acid production and adipogenesis (77). Furthermore, smoking induces hypoxia-inducible factor-1 (HIF-1) expression, which activates HIF-1-dependent adipogenesis in hypoxic conditions (78). Additionally, smoking exacerbates oxidative stress in TAO orbital fibroblasts, leading to the upregulation of fibrosis-related genes such as apolipoprotein J, connective tissue growth factor, and fibronectin. This process is accompanied by increased levels of TGF-β1 and IL-1β (79).

Non-coding RNAs are also implicated in the regulation of TAO orbital tissue fibrosis. Specifically, miR-146a downregulates fibronectin, collagen I α, and α-SMA proteins in TGF-β-induced TAO orbital fibroblasts via SMAD4 and tumor necrosis factor receptor-associated factor 6 pathways (80). Elevated miR-155 levels and decreased miR-146a levels can promote the proliferation of orbital fibroblasts (81). Furthermore, TSH stimulates the proliferation of orbital fibroblasts through the PI3K/Akt pathway as well as miR-146a and miR-155 (82).

Ferroptosis also plays a significant role in anti-fibrosis mechanisms. Triptolide has been shown to alleviate liver fibrosis by inducing ferroptosis in hepatic stellate cells via HO-1 targeting, offering a novel approach to treating liver fibrosis (83). The induction of ferroptosis in activated hepatic stellate cells is emerging as a promising and innovative approach for liver fibrosis therapy (84). However, research has indicated that excessive iron deposition and ferroptosis in the liver exacerbate acetaminophen-induced liver fibrosis in murine models (85). Consequently, additional research is warranted to elucidate the role of ferroptosis in fibrosis. Specifically, in the context of pulmonary fibrosis, stimulation by TGF-β has been shown to upregulate the expression of transferrin receptor protein 1 in both human lung fibroblasts and primary mouse lung fibroblasts (86). This upregulation results in an increased intracellular concentration of Fe^2+^, thereby facilitating the differentiation of fibroblasts into myofibroblasts (87). Consequently, we propose that ferroptosis may regulate tissue fibrosis through its influence on associated signaling pathways and the enhancement of intercellular interactions. This presents a promising target for antifibrotic therapy in TAO. Furthermore, investigating the inducers and underlying mechanisms of ferroptosis could facilitate the development of novel therapeutic strategies.

**Figure 1:**
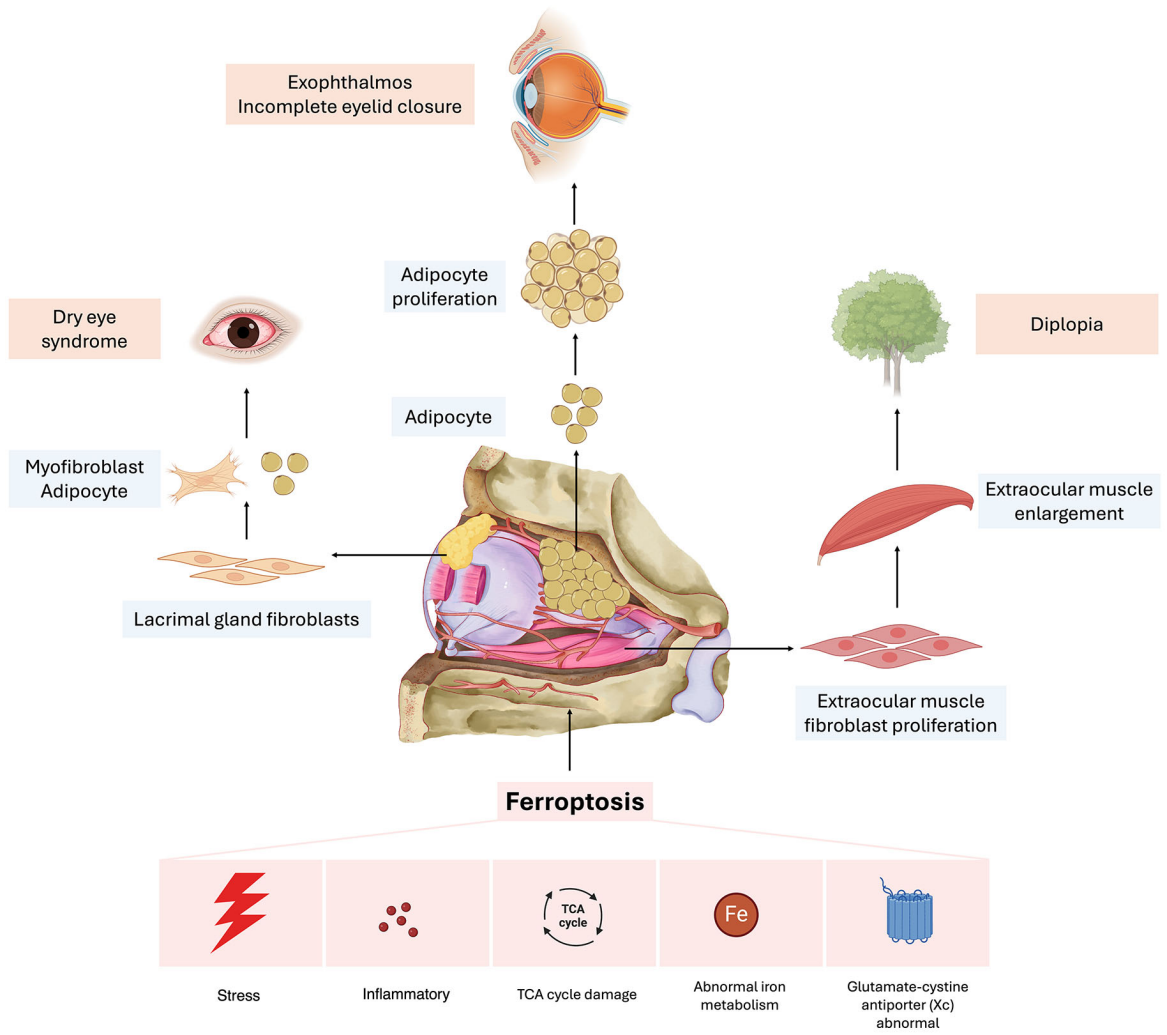
Potential mechanisms of ferroptosis in the progression of thyroid-associated ophthalmopathy. Aberrations in iron metabolism, alongside external stimuli, initiate the inflammatory response. Furthermore, these metabolic irregularities contribute to the process of ferroptosis by enhancing reactive oxygen species (ROS) production and lipid peroxidation through the Fenton reaction. The regulation of insulin-like growth factor 1 (IGF-1) influences the tricarboxylic acid (TCA) cycle and glycolysis, thereby modulating ROS production, which subsequently impacts the incidence of ferroptosis. Moreover, the engagement of diverse inflammatory mediators in the activation of inflammation-associated pathways initiates lipid peroxidation, elevates ROS levels, and induces the formation of inflammatory vesicles. Collectively, these processes contribute to the pathogenesis of ferroptosis. These regulatory mechanisms may play a role in the damage observed in the lacrimal glands, extraocular muscles, and adipose tissues within the orbital regions of patients with thyroid-associated ophthalmopathy (TAO).

In summary, the immunological background of TAO is characterized by a complex interplay of autoimmune responses, involving various immune cells and cytokines, oxidative stress, and ferroptosis (**Figure 1**). Genetic and epigenetic factors further modulate the susceptibility and progression of the disease. Understanding these interrelated mechanisms is essential for identifying new therapeutic targets, underscoring the importance of an integrative treatment approach for this debilitating condition.

### 2.5 The role of ferroptosis in orbital tissue

**Figure 2:**
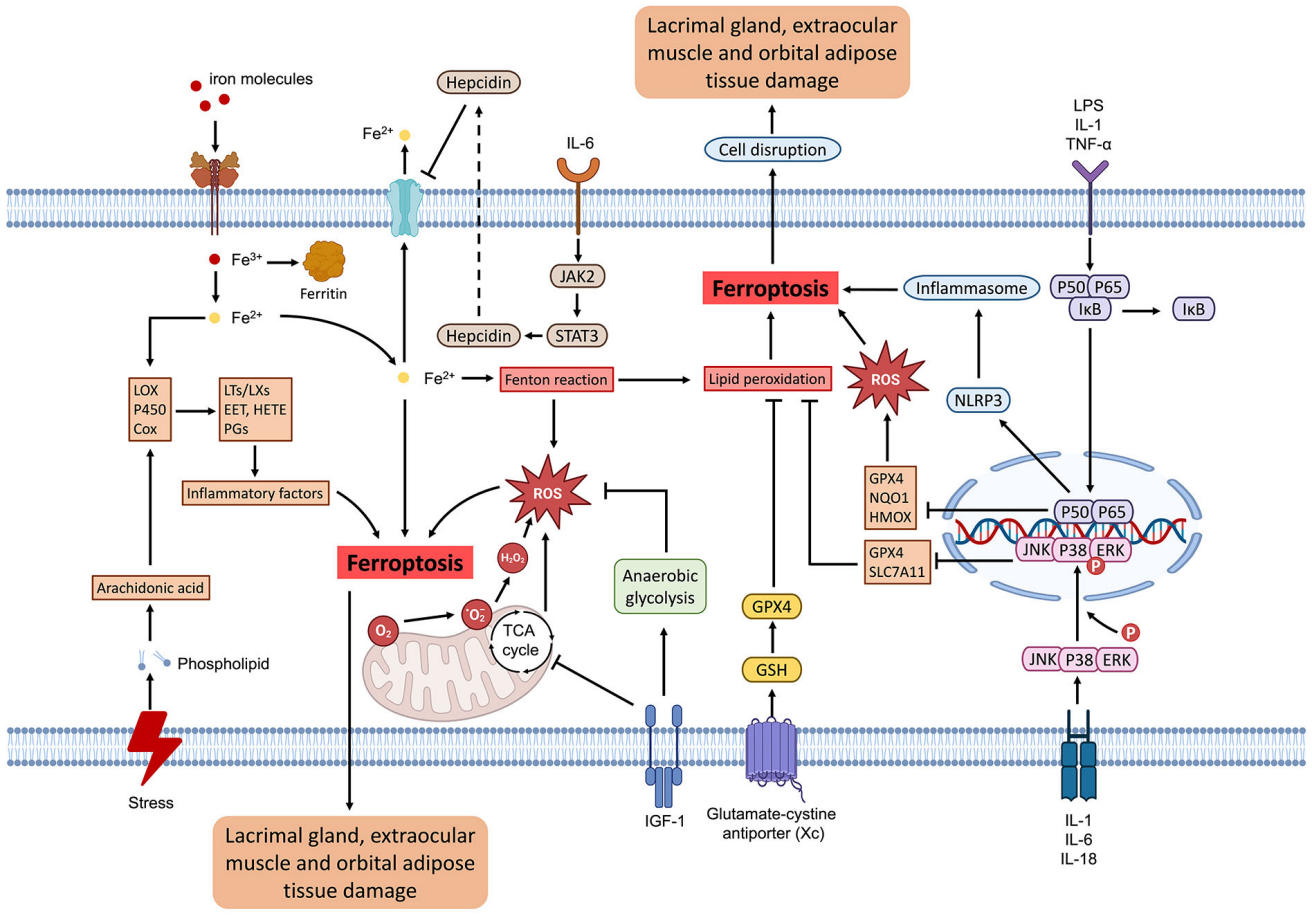
Schematic illustration of orbital tissue damage due to ferroptosis in thyroid-associated ophthalmopathy. Ferroptosis can be triggered by dysregulation in iron metabolism, external stimuli, inflammatory mediators, the tricarboxylic acid (TCA) cycle, and the glutamate-cystine antiporter. In the orbital region, ferroptosis can induce the transformation of lacrimal gland fibroblasts into myofibroblasts and adipocytes, thereby diminishing tear secretion and leading to complications such as dry eye syndrome. The impact on orbital adipose tissue may induce adipose tissue proliferation, culminating in infiltrative proptosis, which subsequently alters the patient’s ocular appearance and causes incomplete eyelid closure. Concurrently, the influence on extraocular muscles may stimulate the proliferation of extraocular muscle fibroblasts, leading to diplopia and other associated complications.

TAO is a multifaceted autoimmune disorder involving various orbital tissues, including extraocular muscles, adipose tissue, and lacrimal glands. Research has identified an age-associated trend in extraocular muscle thickening in TAO patients, with older individuals are more susceptible to posterior orbital extraocular muscle thickening. This thickening can lead to diplopia and restricted ocular motility (88). Furthermore, research indicates that the deprivation of cysteine and/or treatment with erastin can induce ferroptosis, subsequently resulting in the proliferation of orbital fibroblasts (89). In patients with TAO, there is an observed increase in ferroptosis in orbital adipose tissue; however, this does not influence adipocyte proliferation. It is hypothesized that, despite a reduction in GPX4 levels, these adipocytes may exhibit a resemblance to certain tumor cells by maintaining elevated intracellular GSH levels. Consequently, even under conditions of heightened oxidative stress, the residual GSH is essential for neutralizing the substantial amounts of ROS and scavenging lipid peroxides, allowing differentiated adipocytes to resist ferroptosis despite elevated oxidative stress (90). A separate investigation demonstrated that adipocytes have the capacity to secrete fatty acids that promote resistance to ferroptosis in breast cancer cells. This mechanism is reliant on the fatty acid synthase ACSL3, thereby corroborating that breast cancer cells exhibit resistance to ferroptosis when co-cultured with peritumoral adipocytes (91). The function of the lacrimal gland tissue is important for the maintenance of ocular surface health. An investigation into the T-cell immunophenotype of the lacrimal gland in TAO revealed that the inflammation of the lacrimal gland is characterized by a significant infiltration of IFN-γ-producing T helper 1 cells and IL-17A-producing T helper 17 cells. Furthermore, the study demonstrated that IL-17A facilitates the differentiation of lacrimal fibroblasts into either myofibroblasts or adipocytes (92). Furthermore, a study conducted on mice demonstrated that corneal nerve injury induces the upregulation of ferroptosis in lacrimal tissue. This process of ferroptosis subsequently results in damage to the lacrimal tissue, thereby leading to a reduction in tear production (93). In patients with TAO, the synergistic effects of orbital fat accumulation and fibroblast proliferation contribute to infiltrative proptosis, while damage to the lacrimal gland leads to dry eye syndrome. This condition can result in corneal damage due to exposure keratitis, which exacerbates lacrimal gland impairment. Consequently, a self-perpetuating cycle of ocular damage may ensue, potentially leading to further deterioration of ocular health. In conclusion, it is evident that ferroptosis contributes to orbital tissue damage in patients with TAO, leading to orbital fat proliferation, extraocular muscle hypertrophy, and lacrimal gland dysfunction, thereby exacerbating the orbital tissue lesions observed in these patients (**Figure 2**).

## 3 Application of ferroptosis in the treatment of TAO

### 3.1 Potential therapeutic value of ferroptosis inhibitors

Ferroptosis, a form of regulated cell death characterized by iron-dependent lipid peroxidation, has emerged as a promising therapeutic target in various diseases, including TAO (**Table 1**). The inhibition of ferroptosis could potentially mitigate the oxidative stress and cellular damage observed in TAO. Understanding the role of ferroptosis in TAO opens new avenues for therapeutic interventions. Ferroptosis inhibitors can prevent cell death through anti-oxidative stress, anti-fibrosis and blocking lipid peroxidation, and may provide a new approach for the treatment of TAO. For instance, the use of sulfasalazine, a clinically used ferroptosis inducer, has shown promising therapeutic effects in thyroid cancer cells, suggesting its potential application in TAO (94, 95). Additionally, targeting ferroptosis-related pathways, such as the ERK/AP-1 pathway, which has been implicated in the protective effects of lutein on TAO, may further enhance therapeutic outcomes (96). Studies have shown that ferroptosis inhibitors, such as liproxstatin-1 and ferrostatin-1, can effectively reduce lipid peroxidation and cell death in models of oxidative stress-related diseases (97–99). Besides, Selenium is an effective drug for treating TAO and is also a key regulator of ferroptosis, because it reduces hydroperoxy groups of complex lipids and silences lipoxygenases (100, 101). By scavenging lipid peroxyl radicals and preventing the accumulation of toxic lipid peroxides, these inhibitors may reduce inflammation and tissue remodeling in the orbit, key pathological features in TAO (102).

**Table 1:** Possible treatment options for thyroid-associated ophthalmopathy targeting ferroptosis.

Therapy	Mechanism	Targets	Models	References
Sulfasalazine	Blocking lipid peroxidation and ROS accumulation	T-cell originated protein kinase	Clinical patient	(87, 88)
Lutein	Inhibit inflammation and fibrosis	Targeting ERK/AP-1 pathway	Orbital fibroblasts	(89)
Liproxstatin-1 Ferrostatin-1	Scavenging lipid peroxyl radicals and preventing the accumulation of toxic lipid peroxides	GPX4 and SLC7A11	Animal model	(90–92)
Selenium	Reduces hydroperoxy groups of complex lipids and silences lipoxygenases	GPX4	Clinical patient	(93, 94)

### 3.2 Preclinical studies on ferroptosis modulators in TAO treatment

Preclinical studies have provided valuable insights into the potential of ferroptosis modulators in the treatment of TAO. For instance, research on thyroid cancer cells has demonstrated that modulating ferroptosis can influence cell viability and oxidative stress responses. In a study involving anaplastic thyroid cancer cells, it was found that these cells could reduce CD71 levels to increase their tolerance to iron overload, thereby resisting ferroptosis (6). This finding suggests that targeting ferroptosis pathways could be a viable approach to managing TAO, where oxidative stress plays a critical role in disease progression. Additionally, the use of ferroptosis inducers, such as erastin, in combination with iron chelators, has shown promise in reducing oxidative damage and improving cell survival in preclinical models (103). The potential role of ferroptosis in TAO pathogenesis is supported by the observation that ferroptosis-related pathways are activated in the disease. For example, the overexpression of SIRT6 in thyroid cancer has been shown to increase sensitivity to ferroptosis through nuclear receptor coactivator 4-dependent autophagic degradation of ferritin, suggesting a similar mechanism may be at play in TAO (104). Further supporting this hypothesis, differentially expressed ferroptosis-related lncRNAs, such as LINC01140 and ZFHX4-AS1, have been identified in TAO patients (45). These molecular insights highlight the potential of targeting ferroptosis pathways as a therapeutic strategy for TAO, aiming to mitigate oxidative stress and inflammation.

### 3.3 Potential advantages of targeting ferroptosis for therapy

#### 3.3.1 More targeted therapeutic effects

The mechanisms underlying ferroptosis predominantly involve the metabolism of ions and lipid peroxidation, processes intimately associated with oxidative stress in cellular environments related to TAO (105). In TAO, the inflammation and fibrosis of ocular tissues are frequently accompanied by the accumulation of intracellular iron and heightened oxidative stress. Inhibitors of ferroptosis may offer a more efficacious therapeutic strategy by modulating intracellular iron levels and lipid peroxidation, thereby directly targeting the affected tissues (43).

#### 3.3.2 Reduced side effects

Conventional therapeutic approaches, such as high-dose corticosteroid administration, are associated with a range of adverse effects, including weight gain, hyperglycemia, and osteoporosis (106). Conversely, ferroptosis inhibitors may exert a reduced impact on non-target cells, potentially leading to fewer systemic side effects (107).

#### 3.3.3 For patients who do not respond to conventional treatments

Certain patients with TAO exhibit suboptimal responses or develop resistance to conventional treatments such as corticosteroids or radiotherapy (108). In such instances, ferroptosis inhibitors may present novel therapeutic opportunities. By inducing ferroptosis, these agents have the potential to circumvent resistance to standard therapies, thereby providing renewed hope for affected patients (109).

## 4 Challenges and future research directions

### 4.1 Safety considerations in ferroptosis-based TAO treatments

The application of ferroptosis-based therapies in treating TAO presents several safety concerns that must be addressed before clinical implementation. Ferroptosis, characterized by iron-dependent lipid peroxidation, can lead to unintended cytotoxicity in non-target tissues, potentially exacerbating oxidative stress and inflammation (110). The delicate balance between therapeutic efficacy and adverse effects necessitates rigorous preclinical and clinical evaluations. For instance, the use of ferroptosis inducers must be finely tuned to avoid excessive cell death in healthy ocular tissues, which could lead to complications such as vision impairment or loss (111). Additionally, the systemic effects of ferroptosis inducers, particularly their impact on iron metabolism and oxidative stress in other organs, must be carefully monitored to prevent off-target effects (112). The clinical implications of targeting ferroptosis in TAO are significant, given the current limitations of existing therapies. Traditional treatments for TAO, such as corticosteroids and immunosuppressants, often come with substantial side effects and variable efficacy. The introduction of ferroptosis inhibitors and modulators could offer a more targeted approach, potentially reducing the need for invasive procedures like orbital decompression surgery. Moreover, the development of biomarkers to monitor ferroptosis activity in patients with TAO could enhance the precision of treatment strategies, allowing for personalized therapeutic interventions (113). The financial implications associated with ferroptosis inhibitors, as emerging therapeutic modalities, necessitate substantial investment in research, development, and clinical trials. The elevated costs associated with novel pharmaceuticals may constrain their availability within clinical settings. Consequently, exploring the repurposing of existing drugs and the utilization of natural plant extracts could potentially mitigate treatment expenses. Furthermore, the extent of insurance coverage may influence patient acceptance and accessibility to these therapies. Patient acceptance of novel treatments, such as ferroptosis inhibitors, can be challenging due to their recent introduction and the limited knowledge patients may have regarding their efficacy and safety. Consequently, it is essential to enhance patient awareness and comprehension of this innovative therapy. Achieving this may necessitate comprehensive explanations and educational efforts by healthcare providers.

### 4.2 Clinical trial design for ferroptosis modulators

The design of clinical trials for ferroptosis modulators in TAO treatment presents several challenges. One critical aspect is the selection of appropriate biomarkers to monitor ferroptosis activity and therapeutic response. Biomarkers such as lipid peroxidation products and iron levels in ocular tissues could provide insights into the efficacy and safety of ferroptosis-based therapies (114). Furthermore, patient stratification based on genetic and molecular profiles may enhance the precision of these therapies, ensuring that only those likely to benefit are included in the trials (115). Another challenge is the potential variability in ferroptosis sensitivity among patients, which could affect treatment outcomes. Therefore, adaptive trial designs that allow for modifications based on interim results may be necessary to optimize dosing regimens and improve therapeutic efficacy (116).

### 4.3 Long-term efficacy and monitoring

The long-term efficacy of ferroptosis-based treatments for TAO remains an area of active investigation. While preliminary studies have shown promise, the durability of therapeutic effects and the potential for recurrence of TAO symptoms must be evaluated through extended follow-up periods (117). Continuous monitoring for signs of relapse and late-onset adverse effects is crucial to ensure sustained benefits and safety. Additionally, understanding the mechanisms underlying potential resistance to ferroptosis in TAO could inform combination therapies designed enhance and prolong treatment efficacy (107). For instance, combining ferroptosis inducers with immunomodulators or antioxidants may provide synergistic effects, reducing the likelihood of resistance (118).

In conclusion, ferroptosis represents a novel and promising therapeutic target in TAO. However, to successfully integrate this approach into clinical practice, it is essential to address the associated challenges, including safety, trial design, and long-term efficacy. Future research should focus on refining ferroptosis-based strategies to optimize therapeutic benefits while mitigating risks, ultimately improving outcomes for TAO patients.

## 5 Conclusion

TAO presents a complex interplay of immunological and inflammatory processes, with recent studies highlighting the role of ferroptosis in its pathogenesis. Evidence suggests that ferroptosis significantly contributes to the oxidative stress and cellular damage observed in TAO, highlighting new opportunities for therapeutic intervention. Targeting ferroptosis could provide a novel approach, given its close association with iron metabolism and ROS, both of which are involved in the oxidative stress characteristic of TAO. This alignment strengthens the plausibility of ferroptosis as a pivotal mechanism in TAO progression.

Furthermore, distinguishing ferroptosis from other cell death pathways, such as apoptosis and necrosis, offers a more nuanced view of cellular dynamics in TAO and could lead to more specific and effective treatments. However, it is evident that while ferroptosis inhibitors show promise in preclinical models, the translation into clinical practice requires meticulous consideration. The potential therapeutic benefits must be weighed against the safety profiles. Additionally, the design of clinical trials must address the heterogeneity among TAO patients and disease stages to accurately assess the efficacy and safety of ferroptosis-targeted therapies.

In conclusion, ferroptosis represents a compelling therapeutic target in TAO, with its inhibition potentially reducing oxidative damage and inflammation in the disease. Future research should prioritize optimizing ferroptosis inhibitors, ensuring their safety, and validating their clinical effectiveness through rigorous trials. Investigating ferroptosis in TAO not only deepens our understanding of the disease’s underlying mechanisms but also holds significant promise for improving patient outcomes, ushering in a new era in TAO management.

## References

[1] Fang S Lu Y Huang Y Zhou H Fan X. Mechanisms that underly T cell immunity in Graves' Orbitopathy. Front Endocrinol (Lausanne). (2021) 12:648732. doi: 10.3389/fendo.2021.648732 33868176 PMC8049604

[2] Comi S Cosentino G Lanzolla G Menconi F Maglionico MN Posarelli C. Long-term outcome of Graves' orbitopathy following treatment with sirolimus. J Endocrinol Invest. (2024). doi: 10.1007/s40618-024-02470-8 PMC11876206 39373962

[3] Mishra S Maurya VK Kumar S Ankita Kaur A Saxena SK. Clinical management and therapeutic strategies for the thyroid-associated ophthalmopathy: current and future perspectives. Curr Eye Res. (2020) 45:1325–41. doi: 10.1080/02713683.2020.1776331 32567373

[4] Bartalena L Piantanida E Gallo D Lai A Tanda ML. Epidemiology, natural history, risk factors, and prevention of Graves' Orbitopathy. Front Endocrinol (Lausanne). (2020) 11:615993. doi: 10.3389/fendo.2020.615993 33329408 PMC7734282

[5] Zheng J Duan H You S Liang B Chen Y Huang H. Research progress on the pathogenesis of Graves' ophthalmopathy: Based on immunity, noncoding RNA and exosomes. Front Immunol. (2022) 13:952954. doi: 10.3389/fimmu.2022.952954 36081502 PMC9445982

[6] D'Aprile S Denaro S Pavone AM Giallongo S Giallongo C Distefano A. Anaplastic thyroid cancer cells reduce CD71 levels to increase iron overload tolerance. J Transl Med. (2023) 21:780. doi: 10.1186/s12967-023-04664-9 37924062 PMC10625232

[7] Zhang H Wu S Hu S Fan X Song X Feng T. Prediction models of intravenous glucocorticoids therapy response in thyroid eye disease. Eur Thyroid J. (2024) 13:e240122. doi: 10.1530/ETJ-24-0122 39186944 PMC11378126

[8] Rymuza J Pelewicz K Przedlacki J Miśkiewicz P. Therapy with intravenous methylprednisolone pulses is associated with loss of bone microarchitecture in trabecular bone score -assessment among patients with moderate-to-severe Graves' Orbitopathy: A pilot study. Front Endocrinol (Lausanne). (2022) 13:893600. doi: 10.3389/fendo.2022.893600 35909547 PMC9331277

[9] White AM Craig AJ Richie DL Corley C Sadek SM Barton HN. Nicotine is an immunosuppressant: implications for women's health and disease. J Neuroimmunol. (2024) 397:578468. doi: 10.1016/j.jneuroim.2024.578468 39461120 PMC11653054

[10] Tian X Li N Su R Dai C Zhang RA-O. Selenium supplementation may decrease thyroid peroxidase antibody titer via reducing oxidative stress in euthyroid patients with autoimmune thyroiditis. Int J Endocrinol. (2020) 2020:9210572. doi: 10.1155/2020/9210572 32676110 PMC7345605

[11] Negro R Hegedüs L Attanasio R Papini E Winther KH. A 2018 European thyroid association survey on the use of selenium supplementation in Graves' Hyperthyroidism and Graves' Orbitopathy. Eur Thyroid J. (2019) 8:7–15. doi: 10.1159/000494837 30800636 PMC6381891

[12] Douglas RS Kahaly GJ Patel A Sile S Thompson EHZ Perdok R. Teprotumumab for the treatment of active thyroid eye disease. N Engl J Med. (2020) 382:341–52. doi: 10.1056/NEJMoa1910434 31971679

[13] Jain AA-O Jaru-Ampornpan P Douglas RS. Thyroid eye disease: Redefining its management-A review. Clin Exp Ophthalmol. (2021) 49:203–11. doi: 10.1111/ceo.13899 33484076

[14] Vargas-Uricoechea H. Molecular mechanisms in autoimmune thyroid disease. Cells. (2023) 16:918. doi: 10.3390/cells12060918 PMC10047067 36980259

[15] Morshed SA Ma R Latif R Davies TF. How one TSH receptor antibody induces thyrocyte proliferation while another induces apoptosis. J Autoimmun. (2013) 47:17–24. doi: 10.1016/j.jaut.2013.07.009 23958398 PMC3872079

[16] Smith TJ Hegedüs L Douglas RS. Role of insulin-like growth factor-1 (IGF-1) pathway in the pathogenesis of Graves' orbitopathy. Best Pract Res Clin Endocrinol Metab. (2012) 26:291–302. doi: 10.1016/j.beem.2011.10.002 22632366 PMC3712747

[17] Zhang P Zhu H. Cytokines in thyroid-associated ophthalmopathy. J Immunol Res. (2022) 2022:2528046. doi: 10.1155/2022/2528046 36419958 PMC9678454

[18] Kardalas E Maraka S Papagianni M Paltoglou GA-O Siristatidis CA-O Mastorakos G. TGF-β Physiology as a novel therapeutic target regarding autoimmune thyroid diseases: where do we stand and what to expect. LID - 10.3390/medicina57060621 [doi] LID - 621. Medicina (Kaunas). (2021) 57:621. doi: 10.3390/medicina57060621 34198624 PMC8232149

[19] Chiu H Wu S Tsai C. The role of fibrogenesis and extracellular matrix proteins in the pathogenesis of Graves' Ophthalmopathy. Int J Mol Sci. (2024) 25:3288. doi: 10.3390/ijms25063288 38542262 PMC10970309

[20] Yang IH Rose GE Ezra DG Bailly M. Macrophages promote a profibrotic phenotype in orbital fibroblasts through increased hyaluronic acid production and cell contractility. Sci Rep. (2019) 9:9622. doi: 10.1038/s41598-019-46075-1 31270379 PMC6610127

[21] Fei Y Ding Y. The role of ferroptosis in neurodegenerative diseases. Front Cell Neurosci. (2024) 18:1475934. doi: 10.3389/fncel.2024.1475934 39473490 PMC11518764

[22] Zhou D Yang Y Han R He J Liu D Xia W. Ferroptosis and its potential determinant role in myocardial susceptibility to ischemia/reperfusion injury in diabetes. Rev Cardiovasc Med. (2024) 25:360. doi: 10.31083/j.rcm2510360 39484139 PMC11522832

[23] Wang S Guo Q Zhou L Xia X. Ferroptosis: A double-edged sword. Cell Death discov. (2024) 10:265. doi: 10.1038/s41420-024-02037-9 38816377 PMC11139933

[24] Conrad M Friedmann Angeli JP. Glutathione peroxidase 4 (Gpx4) and ferroptosis: what's so special about it? Mol Cell Oncol. (2015) 2:e995047. doi: 10.4161/23723556.2014.995047 27308484 PMC4905320

[25] Yang Y Lin Y Han Z Wang B Zheng W Wei L. Ferroptosis: a novel mechanism of cell death in ophthalmic conditions. Front Immunol. (2024) 15:1440309. doi: 10.3389/fimmu.2024.1440309 38994366 PMC11236620

[26] Lei G Mao C Yan Y Zhuang L Gan B. Ferroptosis, radiotherapy, and combination therapeutic strategies. Protein Cell. (2021) 12:836–57. doi: 10.1007/s13238-021-00841-y PMC8563889 33891303

[27] Lu Q Lu X Zhang Y Huang W Zhou H Li T. Recent advances in ferroptosis and therapeutic strategies for glioblastoma. Front Mol biosciences. (2022) 9:1068437. doi: 10.3389/fmolb.2022.1068437 PMC9880056 36710875

[28] Tsai CC Wu SB Cheng CY Kao SC Kau HC Lee SM. Increased response to oxidative stress challenge in Graves' ophthalmopathy orbital fibroblasts. Mol Vis. (2011) 17:2782–8. PMC3209425 22065933

[29] Ma C Li H Liu W Lu S Li X Chen J. Therapeutic effect of gypenosides on antioxidant stress injury in orbital fibroblasts of Graves' Orbitopathy. J Immunol Res. (2022) 2022:4432584. doi: 10.1155/2022/4432584 36157877 PMC9499793

[30] Hung CT Tsai YW Wu YS Yeh CF Yang KC. The novel role of ER protein TXNDC5 in the pathogenesis of organ fibrosis: mechanistic insights and therapeutic implications. J Biomed Sci. (2022) 29:63. doi: 10.1186/s12929-022-00850-x 36050716 PMC9438287

[31] Ko J Kim JY Kyoung Chae M Jig Lee E Sook Yoon J. PERK mediates oxidative stress and adipogenesis in Graves' orbitopathy pathogenesis. J Mol Endocrinol. (2021) 66:313–23. doi: 10.1530/jme-21-0057 33870911

[32] Ekronarongchai S Palaga T Saonanon P Pruksakorn V Hirankarn N van Hagen PM. Histone deacetylase 4 controls extracellular matrix production in orbital fibroblasts from Graves' Ophthalmopathy patients. Thyroid. (2021) 31:1566–76. doi: 10.1089/thy.2020.0948 34235979

[33] Chen Y Tang R Xiong W Zhang F Wang N Xie B. RNA aptamers with specific binding affinity to CD40 (CD40Apt) represents a promising antagonist of the CD40-CD40L signaling for thyroid-associated ophthalmopathy (TAO) treatment in mouse. J Transl Med. (2023) 21:396. doi: 10.1186/s12967-023-04217-0 37331977 PMC10278284

[34] Ai Y Meng Y Yan B Zhou Q Wang X. The biochemical pathways of apoptotic, necroptotic, pyroptotic, and ferroptotic cell death. Mol Cell. (2024) 84:170–79. doi: 10.1016/j.molcel.2023.11.040 38181758

[35] Woo Y Lee HJ Jung YM Jung YJ. Regulated necrotic cell death in alternative tumor therapeutic strategies. Cells. (2020) 9:2709. doi: 10.3390/cells9122709 33348858 PMC7767016

[36] Zheng J Conrad M. The metabolic underpinnings of ferroptosis. Cell Metab. (2020) 32:920–37. doi: 10.1016/j.cmet.2020.10.011 33217331

[37] Mao C Liu X Zhang Y Lei G Yan Y Lee H. DHODH-mediated ferroptosis defence is a targetable vulnerability in cancer. Nature. (2021) 593:586–90. doi: 10.1038/s41586-021-03539-7 PMC8895686 33981038

[38] Kraft VAN Bezjian CT Pfeiffer S Ringelstetter L Müller C Zandkarimi F. GTP cyclohydrolase 1/Tetrahydrobiopterin counteract ferroptosis through lipid remodeling. ACS Cent science. (2020) 6:41–53. doi: 10.1021/acscentsci.9b01063 PMC6978838 31989025

[39] Yang WS SriRamaratnam R Welsch ME Shimada K Skouta R Viswanathan VS. Regulation of ferroptotic cancer cell death by GPX4. Cell. (2014) 156:317–31. doi: 10.1016/j.cell.2013.12.010 PMC4076414 24439385

[40] Li J Cao F Yin HL Huang ZJ Lin ZT Mao N. Ferroptosis: past, present and future. Cell Death Dis. (2020) 11:88. doi: 10.1038/s41419-020-2298-2 32015325 PMC6997353

[41] Gan B. Mitochondrial regulation of ferroptosis. J Cell Biol. (2021) 220:e202105043. doi: 10.1083/jcb.202105043 34328510 PMC8329737

[42] Navarro E Gonzalez-Lafuente L Pérez-Liébana I Buendia I López-Bernardo E Sánchez-Ramos C. Heme-oxygenase I and PCG-1α Regulate mitochondrial biogenesis via microglial activation of alpha7 nicotinic acetylcholine receptors using PNU282987. Antioxid Redox Signal. (2017) 27:93–105. doi: 10.1089/ars.2016.6698 27554853

[43] Ye Y Dai L Mugaanyi J Fu W Hu F. Novel insights into the pathogenesis of thyroid eye disease through ferroptosis-related gene signature and immune infiltration analysis. Aging (Albany NY). (2024) 16:6008–34. doi: 10.18632/aging.205685 PMC11042930 38536014

[44] Gao Y Li W. Mechanisms of immune-related differentially expressed genes in thyroid-associated ophthalmopathy based on the GEO database. Ann Trans Med. (2022) 10:926. doi: 10.21037/atm-22-3470 PMC9511181 36172114

[45] Chen S Diao J Yue Z Wei R. Identification and validation of ferroptosis-related genes and immune cell infiltration in thyroid associated ophthalmopathy. Front Genet. (2023) 14:1118391. doi: 10.3389/fgene.2023.1118391 37021001 PMC10067720

[46] Shin HR Cho WK Baek IC Lee NY Lee YJ Kim SK. Polymorphisms of IRAK1 gene on X chromosome is associated with Hashimoto thyroiditis in Korean children. Endocrinology. (2020) 161:bqaa088. doi: 10.1210/endocr/bqaa088 32498091

[47] Rajesh Y Kanneganti TD. Innate immune cell death in neuroinflammation and Alzheimer's disease. Cells. (2022) 11:1885. doi: 10.3390/cells11121885 35741014 PMC9221514

[48] Liu W Ma C Li HY Yuan SS Li KJ. Tea polyphenols reduce inflammation of orbital fibroblasts in Graves' Ophthalmopathy via the NF-κB/NLRP3 pathway. Curr Med science. (2023) 43:123–29. doi: 10.1007/s11596-023-2708-7 36821039

[49] Massagué J Sheppard D. TGF-β signaling in health and disease. Cell. (2023) 186:4007–37. doi: 10.1016/j.cell.2023.07.036 PMC10772989 37714133

[50] Li K Li H Xu W Liu W Du Y He JF. Research on the potential mechanism of gypenosides on treating thyroid-associated ophthalmopathy based on network pharmacology. Med Sci Monit. (2019) 25:4923–32. doi: 10.12659/msm.917299 PMC6621796 31268042

[51] Li H Min J Yang Y Suo W Wang W Tian J. TMEM2 inhibits the development of Graves' orbitopathy through the JAK-STAT signaling pathway. J Biol Chem. (2024) 300:105607. doi: 10.1016/j.jbc.2023.105607 38159864 PMC10839445

[52] Zhou M Lin B Wu P Ke Y Huang S Zhang F. SOX9 induces orbital fibroblast activation in thyroid eye disease via MAPK/ERK1/2 pathway. Invest Ophthalmol Vis Sci. (2024) 65:25. doi: 10.1167/iovs.65.2.25 PMC10866156 38345552

[53] Kim DW Taneja K Hoang T Santiago CP McCulley TJ Merbs SL. Transcriptomic profiling of control and thyroid-associated orbitopathy (TAO) orbital fat and TAO orbital fibroblasts undergoing adipogenesis. Invest Ophthalmol Vis Sci. (2021) 62:24. doi: 10.1167/iovs.62.9.24 PMC8297424 34269815

[54] Li B Smith TJ. PI3K/AKT pathway mediates induction of IL-1RA by TSH in fibrocytes: modulation by PTEN. J Clin Endocrinol Metab. (2014) 99:3363–72. doi: 10.1210/jc.2014-1257 PMC4154109 24840811

[55] Jiang W Zhang LX Tan XY Yu P Dong M. Inflammation and histone modification in chronic pain. Front Immunol. (2022) 13:1087648. doi: 10.3389/fimmu.2022.1087648 36713369 PMC9880030

[56] Radziszewski M Kuś A Bednarczuk T. Genotype-phenotype correlations in Graves' disease. Best Pract Res Clin Endocrinol Metab. (2023) 37:101745. doi: 10.1016/j.beem.2023.101745 36828713

[57] Li Z Zhang C Liu Y Wang F Zhao B Yang J. Diagnostic and predictive values of ferroptosis-related genes in child sepsis. Front Immunol. (2022) 13:881914. doi: 10.3389/fimmu.2022.881914 35844488 PMC9281550

[58] Wang T Fu X Chen Q Patra JK Wang D Wang Z. Arachidonic acid metabolism and kidney inflammation. Int J Mol Sci. (2019) 20:3683. doi: 10.3390/ijms20153683 31357612 PMC6695795

[59] Yao F Deng Y Zhao Y Mei Y Zhang Y Liu X. A targetable LIFR-NF-κB-LCN2 axis controls liver tumorigenesis and vulnerability to ferroptosis. Nat Commun. (2021) 12:7333. doi: 10.1038/s41467-021-27452-9 34921145 PMC8683481

[60] Tsurusaki S Tsuchiya Y Koumura T Nakasone M Sakamoto T Matsuoka M. Hepatic ferroptosis plays an important role as the trigger for initiating inflammation in nonalcoholic steatohepatitis. Cell Death Dis. (2019) 10:449. doi: 10.1038/s41419-019-1678-y 31209199 PMC6579767

[61] Yan N Xu Z Qu C Zhang J. Dimethyl fumarate improves cognitive deficits in chronic cerebral hypoperfusion rats by alleviating inflammation, oxidative stress, and ferroptosis via NRF2/ARE/NF-κB signal pathway. Int Immunopharmacol. (2021) 98:107844. doi: 10.1016/j.intimp.2021.107844 34153667

[62] Barrat FJ Crow MK Ivashkiv LB. Interferon target-gene expression and epigenomic signatures in health and disease. Nat Immunol. (2019) 20:1574–83. doi: 10.1038/s41590-019-0466-2 PMC7024546 31745335

[63] Kong R Wang N Han W Bao W Lu J. IFNγ-mediated repression of system xc(-) drives vulnerability to induced ferroptosis in hepatocellular carcinoma cells. J Leukoc Biol. (2021) 110:301–14. doi: 10.1002/jlb.3ma1220-815rrr 34318944

[64] Wang W Green M Choi JE Gijón M Kennedy PD Johnson JK. CD8(+) T cells regulate tumour ferroptosis during cancer immunotherapy. Nature. (2019) 569:270–74. doi: 10.1038/s41586-019-1170-y PMC6533917 31043744

[65] Wei TT Zhang MY Zheng XH Xie TH Wang W Zou J. Interferon-γ induces retinal pigment epithelial cell Ferroptosis by a JAK1-2/STAT1/SLC7A11 signaling pathway in Age-related Macular Degeneration. FEBS J. (2022) 289:1968–83. doi: 10.1111/febs.16272 34741776

[66] Li J Li L Zhang Z Chen P Shu H Yang C. Ferroptosis: an important player in the inflammatory response in diabetic nephropathy. Front Immunol. (2023) 14:1294317. doi: 10.3389/fimmu.2023.1294317 38111578 PMC10725962

[67] Yao L Kan EM Lu J Hao A Dheen ST Kaur C. Toll-like receptor 4 mediates microglial activation and production of inflammatory mediators in neonatal rat brain following hypoxia: role of TLR4 in hypoxic microglia. J Neuroinflammation. (2013) 10:23. doi: 10.1186/1742-2094-10-23 23388509 PMC3575244

[68] Chen Z Wang W Abdul Razak SR Han T Ahmad NH Li X. Ferroptosis as a potential target for cancer therapy. Cell Death Dis. (2023) 14:460. doi: 10.1038/s41419-023-05930-w 37488128 PMC10366218

[69] Zhu K Zhu X Sun S Yang W Liu S Tang Z. Inhibition of TLR4 prevents hippocampal hypoxic-ischemic injury by regulating ferroptosis in neonatal rats. Exp Neurol. (2021) 345:113828. doi: 10.1016/j.expneurol.2021.113828 34343528

[70] Liu N Liang Y Wei T Zou L Huang X Kong L. The role of ferroptosis mediated by NRF2/ERK-regulated ferritinophagy in CdTe QDs-induced inflammation in macrophage. J Hazard Mater. (2022) 436:129043. doi: 10.1016/j.jhazmat.2022.129043 35525219

[71] Smith TJ Bahn RS Gorman CA. Connective tissue, glycosaminoglycans, and diseases of the thyroid. Endocr Rev. (1989) 10:366–91. doi: 10.1210/edrv-10-3-366 2673756

[72] Smith TJ Janssen JA. Building the case for insulin-like growth factor receptor-I involvement in thyroid-associated ophthalmopathy. Front Endocrinol (Lausanne). (2016) 7:167. doi: 10.3389/fendo.2016.00167 28096798 PMC5206614

[73] Frangogiannis N. Transforming growth factor-β in tissue fibrosis. J Exp Med. (2020) 217:e20190103. doi: 10.1084/jem.20190103 32997468 PMC7062524

[74] Robertson IB Horiguchi M Zilberberg L Dabovic B Hadjiolova K Rifkin DB. Latent TGF-β-binding proteins. Matrix Biol. (2015) 47:44–53. doi: 10.1016/j.matbio.2015.05.005 25960419 PMC4844006

[75] Budi EH Duan D Derynck R. Transforming growth factor-β Receptors and smads: regulatory complexity and functional versatility. Trends Cell Biol. (2017) 27:658–72. doi: 10.1016/j.tcb.2017.04.005 28552280

[76] Finnson KW Almadani Y Philip A. Non-canonical (non-SMAD2/3) TGF-β signaling in fibrosis: Mechanisms and targets. Semin Cell Dev Biol. (2020) 101:115–22. doi: 10.1016/j.semcdb.2019.11.013 31883994

[77] Cawood TJ Moriarty P O'Farrelly C O'Shea D. Smoking and thyroid-associated ophthalmopathy: A novel explanation of the biological link. J Clin Endocrinol Metab. (2007) 92:59–64. doi: 10.1210/jc.2006-1824 17047020

[78] Görtz GE Horstmann M Aniol B Reyes BD Fandrey J Eckstein A. Hypoxia-dependent HIF-1 activation impacts on tissue remodeling in Graves' Ophthalmopathy-implications for smoking. J Clin Endocrinol Metab. (2016) 101:4834–42. doi: 10.1210/jc.2016-1279 27610652

[79] Kau HC Wu SB Tsai CC Liu CJ Wei YH. Cigarette smoke extract-induced oxidative stress and fibrosis-related genes expression in orbital fibroblasts from patients with Graves' Ophthalmopathy. Oxid Med Cell Longev. (2016) 2016:4676289. doi: 10.1155/2016/4676289 27340508 PMC4909929

[80] Jang SY Park SJ Chae MK Lee JH Lee EJ Yoon JS. Role of microRNA-146a in regulation of fibrosis in orbital fibroblasts from patients with Graves' orbitopathy. Br J Ophthalmol. (2018) 102:407–14. doi: 10.1136/bjophthalmol-2017-310723 29101123

[81] Li K Du Y Jiang BL He JF. Increased microRNA-155 and decreased microRNA-146a may promote ocular inflammation and proliferation in Graves' ophthalmopathy. Med Sci Monit. (2014) 20:639–43. doi: 10.12659/msm.890686 PMC3999163 24743332

[82] Woeller CF Roztocil E Hammond C Feldon SE. TSHR Signaling Stimulates Proliferation Through PI3K/Akt and Induction of miR-146a and miR-155 in Thyroid Eye Disease Orbital Fibroblasts. Invest Ophthalmol Vis Sci. (2019) 60:4336–45. doi: 10.1167/iovs.19-27865 PMC6798326 31622470

[83] Luo P Liu D Zhang Q Yang F Wong YK Xia F. Celastrol induces ferroptosis in activated HSCs to ameliorate hepatic fibrosis via targeting peroxiredoxins and HO-1. Acta Pharm Sinica B. (2022) 12:2300–14. doi: 10.1016/j.apsb.2021.12.007 PMC9136576 35646542

[84] Zhang Z Guo M Li Y Shen M Kong D Shao J. RNA-binding protein ZFP36/TTP protects against ferroptosis by regulating autophagy signaling pathway in hepatic stellate cells. Autophagy. (2020) 16:1482–505. doi: 10.1080/15548627.2019.1687985 PMC7469536 31679460

[85] Yu Y Jiang L Wang H Shen Z Cheng Q Zhang P. Hepatic transferrin plays a role in systemic iron homeostasis and liver ferroptosis. Blood. (2020) 136:726–39. doi: 10.1182/blood.2019002907 PMC7414596 32374849

[86] Pei Z Qin Y Fu X Yang F Huo F Liang X. Inhibition of ferroptosis and iron accumulation alleviates pulmonary fibrosis in a bleomycin model. Redox Biol. (2022) 57:102509. doi: 10.1016/j.redox.2022.102509 36302319 PMC9614651

[87] Jian J Wang D Xiong Y Wang J Zheng Q Jiang Z. Puerarin alleviated oxidative stress and ferroptosis during renal fibrosis induced by ischemia/reperfusion injury via TLR4/Nox4 pathway in rats. Acta Cir Bras. (2023) 38:e382523. doi: 10.1590/acb382523 37556718 PMC10403246

[88] Neag EJ Smith TJ. 2021 update on thyroid-associated ophthalmopathy. J Endocrinol Invest. (2022) 45:235–59. doi: 10.1007/s40618-021-01663-9 PMC9455782 34417736

[89] Su Y Liu X Fang S Huang Y Li Y Zhong S. Age-related difference in extraocular muscles and its relation to clinical manifestations in an ethnically homogenous group of patients with Graves' orbitopathy. Graefes Arch Clin Exp Ophthalmol. (2022) 260:583–89. doi: 10.1007/s00417-021-05377-9 34477926

[90] Luo C Sun J Liu D Sun B Miao L Musetti S. Self-assembled redox dual-responsive prodrug-nanosystem formed by single thioether-bridged paclitaxel-fatty acid conjugate for cancer chemotherapy. Nano Lett. (2016) 16:5401–08. doi: 10.1021/acs.nanolett.6b01632 PMC5541379 27490088

[91] Xie Y Wang B Zhao Y Tao Z Wang Y Chen G. Mammary adipocytes protect triple-negative breast cancer cells from ferroptosis. J Hematol Oncol. (2022) 15:72. doi: 10.1186/s13045-022-01297-1 35659320 PMC9164506

[92] Huang Y Wu Y Zhang S Lu Y Wang Y Liu X. Immunophenotype of lacrimal glands in Graves Orbitopathy: implications for the pathogenesis of Th1 and Th17 immunity. Thyroid. (2022) 32:949–61. doi: 10.1089/thy.2021.0671 35469435

[93] Liu X Cui Z Chen X Li Y Qiu J Huang Y. Ferroptosis in the lacrimal gland is involved in dry eye syndrome induced by corneal nerve severing. Invest Ophthalmol Vis Sci. (2023) 64:27. doi: 10.1167/iovs.64.7.27 PMC10281063 37326593

[94] Wang Z Wu S Zhu C Shen J. The role of ferroptosis in esophageal cancer. Cancer Cell Int. (2022) 22:266. doi: 10.1186/s12935-022-02685-w 35999642 PMC9396912

[95] Boskovic O Medenica S Radojevic N Zarkovic M. Etanercept in the treatment of Graves' ophthalmopathy with primary hypothyroidism and rheumatoid arthritis. Cent Eur J Immunol. (2019) 44:463–65. doi: 10.5114/ceji.2019.92803 PMC7050053 32140060

[96] Hei X Lin B Wu P Li X Mao Z Huang S. Lutein targeting orbital fibroblasts attenuates fibrotic and inflammatory effects in thyroid-associated ophthalmopathy. Exp Eye Res. (2023) 232:109515. doi: 10.1016/j.exer.2023.109515 37207866

[97] Zilka O Shah R Li B Friedmann Angeli JP Griesser M Conrad M. On the mechanism of cytoprotection by ferrostatin-1 and liproxstatin-1 and the role of lipid peroxidation in ferroptotic cell death. ACS Cent science. (2017) 3:232–43. doi: 10.1021/acscentsci.7b00028 PMC5364454 28386601

[98] Skouta R Dixon SJ Wang J Dunn DE Orman M Shimada K. Ferrostatins inhibit oxidative lipid damage and cell death in diverse disease models. J Am Chem Soc. (2014) 136:4551–6. doi: 10.1021/ja411006a PMC3985476 24592866

[99] Liu CQ Liu XY Ouyang PW Liu Q Huang XM Xiao F. Ferrostatin-1 attenuates pathological angiogenesis in oxygen-induced retinopathy via inhibition of ferroptosis. Exp Eye Res. (2023) 226:109347. doi: 10.1016/j.exer.2022.109347 36502924

[100] Chung CW Jung KY Jung EH Lee MJ Park YJ Lee JK. Efficacy of selenium supplementation for mild-to-moderate Graves' ophthalmopathy in a selenium-sufficient area (SeGOSS trial): study protocol for a phase III, multicenter, open-label, randomized, controlled intervention trial. Trials. (2023) 24:272. doi: 10.1186/s13063-023-07282-4 37060084 PMC10103450

[101] Conrad M Proneth B. Selenium: tracing another essential element of ferroptotic cell death. Cell Chem Biol. (2020) 27:409–19. doi: 10.1016/j.chembiol.2020.03.012 32275866

[102] Shen L Wang X Zhai C Chen Y. Ferroptosis: A potential therapeutic target in autoimmune disease (Review). Exp Ther Med. (2023) 26:368. doi: 10.3892/etm.2023.12067 37408857 PMC10318600

[103] Ma R Gan L Guo J Peng Z Wu J Harrison AR. Insights into ferroptosis: targeting glycolysis to treat Graves' Orbitopathy. J Clin Endocrinol Metab. (2022) 107:1994–2003. doi: 10.1210/clinem/dgac163 35303084

[104] Yang Z Huang R Wang Y Guan Q Li D Wu Y. SIRT6 drives sensitivity to ferroptosis in anaplastic thyroid cancer through NCOA4-dependent autophagy. Am J Cancer Res. (2023) 13:464–74. PMC9989618 36895980

[105] de Souza IA-O Ramalho MCC Guedes CA-O Osawa IYA Monteiro LKS Gomes LR. Ferroptosis modulation: potential therapeutic target for glioblastoma treatment. LID - 10.3390/ijms23136879 [doi] LID - 6879. Int J Mol Sci. (2022) 23:6879. doi: 10.3390/ijms23136879 35805884 PMC9266903

[106] Pofi R Caratti G Ray DW Tomlinson JA-O. Treating the side effects of exogenous glucocorticoids; can we separate the good from the bad? Endocr Rev. (2023) 44:975–1011. doi: 10.1210/endrev/bnad016 37253115 PMC10638606

[107] Zhou Q Meng Y Li D Yao L Le J Liu Y. Ferroptosis in cancer: From molecular mechanisms to therapeutic strategies. Signal transduction targeted Ther. (2024) 9:55. doi: 10.1038/s41392-024-01769-5 PMC10920854 38453898

[108] Smith TJ Bartalena L. Will biological agents supplant systemic glucocorticoids as the first-line treatment for thyroid-associated ophthalmopathy? Eur J Endocrinol. (2019) 181:D27–43. doi: 10.1530/EJE-19-0389 PMC7398270 31370005

[109] Qi X Wan Z Jiang B Ouyang Y Feng W Zhu H. Inducing ferroptosis has the potential to overcome therapy resistance in breast cancer. Front Immunol. (2022) 13:1038225. doi: 10.3389/fimmu.2022.1038225 36505465 PMC9730886

[110] Yu Y Yan Y Niu F Wang Y Chen X Su G. Ferroptosis: a cell death connecting oxidative stress, inflammation and cardiovascular diseases. Cell Death discov. (2021) 7:193. doi: 10.1038/s41420-021-00579-w 34312370 PMC8313570

[111] Zhang J Sheng S Wang W Dai J Zhong Y Ren J. Molecular mechanisms of iron mediated programmed cell death and its roles in eye diseases. Front Nutr. (2022) 9:844757. doi: 10.3389/fnut.2022.844757 35495915 PMC9038536

[112] Feng S Tang D Wang Y Li X Bao H Tang C. The mechanism of ferroptosis and its related diseases. Mol biomed. (2023) 4:33. doi: 10.1186/s43556-023-00142-2 37840106 PMC10577123

[113] Yuan M He Q Xiang W Deng Y Lin S Zhang R. Natural compounds efficacy in Ophthalmic Diseases: A new twist impacting ferroptosis. Biomed Pharmacother. (2024) 172:116230. doi: 10.1016/j.biopha.2024.116230 38350366

[114] Zhang F Yan Y Cai Y Liang Q Liu Y Peng B. Current insights into the functional roles of ferroptosis in musculoskeletal diseases and therapeutic implications. Front Cell Dev Biol. (2023) 11:1112751. doi: 10.3389/fcell.2023.1112751 36819098 PMC9936329

[115] Fujihara KM Zhang BZ Clemons NJ. Opportunities for ferroptosis in cancer therapy. Antioxidants (Basel Switzerland). (2021) 10:986. doi: 10.3390/antiox10060986 34205617 PMC8235304

[116] Xiang D Zhou L Yang R Yuan F Xu Y Yang Y. Advances in ferroptosis-inducing agents by targeted delivery system in cancer therapy. Int J nanomed. (2024) 19:2091–112. doi: 10.2147/ijn.S448715 PMC10929151 38476278

[117] Lippmann J Petri K Fulda S Liese J. Redox modulation and induction of ferroptosis as a new therapeutic strategy in hepatocellular carcinoma. Transl Oncol. (2020) 13:100785. doi: 10.1016/j.tranon.2020.100785 32416440 PMC7283515

[118] Sun J Huang L Wang J Hu Y Wang W Zhu H. Programmed cell death in autoimmune diseases: ferroptosis. Ann Biol Clin (Paris). (2024) 82:33–42. doi: 10.1684/abc.2024.1866 38638017

